# Editorial Comment from Dr Anan to subcapsular renal hematoma after ureterorenoscopy

**DOI:** 10.1002/iju5.12480

**Published:** 2022-05-22

**Authors:** Go Anan

**Affiliations:** ^1^ Department of Urology Yotsuya Medical Cube Tokyo Japan

Ureteroscopy (URS) is a minimally invasive surgery for treating urolithiasis. The number of URS procedures for treating urolithiasis has increased. Common complications of URS include urinary tract infection, hematuria, ureteral mucosal damage, and ureteral stenosis. Most complications of URS are minor; however, some complications, such as sepsis, urinoma, and ureteral rupture, are severe.^1^ URS has been reported to increase the probability of postoperative urinary tract infection because of prolonged operative time and rupture of the renal pelvis due to increased urinary tract perfusion pressure.^2^ Therefore, it is essential to know in detail how to prevent and treat the complications of URS.

Harada *et al*. reported a case of subcapsular hematoma after URS.^3^ They treated the middle and lower calyx stones, measuring 36 mm in diameter, of the right kidney in 2 h by URS. However, the stones could not be entirely removed. A large stone was fragmented and not properly extracted, resulting in stone street formation and hydronephrosis despite postoperative ureteral stent placement. Here, subcapsular hematoma occurred as a postoperative complication. The subcapsular hematoma was caused because of increased pressure in the renal pelvis due to a temporal increase in perfusion pressure or postoperative ureteral obstruction due to stone street. Subcapsular renal hematoma after URS is an uncommon complication but should be considered.^4^

In this case, contrast‐enhanced computed tomography was conducted on postoperative day 5, despite fever and right lumbar back pain on postoperative day 1. Ultrasonography is a simple test; therefore, it is crucial to promptly conduct it. Additionally, lumbar back pain on the diseased side after URS should be considered for hydronephrosis, ureteral stent obstruction, pyelonephrosis, or urinoma. When URS is conducted on large‐sized stones, a staged procedure is required. In the staged URS, it is crucial to strategically select the areas to be fragmented and actively retrieved to reduce postoperative complications, such as stone street. One method of active retrieval is the one‐surgeon basketing technique.^5^ This case shows the need to avoid excessive intraoperative increase in intrarenal pressure and to strategically fragment and retrieve large renal stones without creating a postoperative stone street.

## Conflict of interest

The author declares no conflict of interest.
